# Novel viruses detected in bats in the Republic of Korea

**DOI:** 10.1038/s41598-020-77307-4

**Published:** 2020-11-20

**Authors:** Sook-Young Lee, Chul-Un Chung, Jun Soo Park, Jae-Ku Oem

**Affiliations:** 1 Laboratory of Veterinary Infectious Diseases, College of Veterinary Medicine, Jeonbuk National University, Iksan, Chonbuk Republic of Korea; 2 Department of Life Science, Dongguk University, Gyeongju, Republic of Korea; 3 Department of Veterinary Infectious Diseases, College of Veterinary Medicine, Jeonbuk National University, Iksan, Republic of Korea

**Keywords:** Computational biology and bioinformatics, Microbiology

## Abstract

Bats are natural reservoirs for potential zoonotic viruses. In this study, next-generation sequencing was performed to obtain entire genome sequences of picornavirus from a picornavirus-positive bat feces sample (16BF77) and to explore novel viruses in a pooled bat sample (16BP) from samples collected in South Korea, 2016. Fourteen mammalian viral sequences were identified from 16BF77 and 29 from 16BP, and verified by RT-PCR. The most abundant virus in 16BF77 was picornavirus. Highly variable picornavirus sequences encoding 3D^pol^ were classified into genera *Kobuvirus*, *Shanbavirus*, and an unassigned group within the family *Picornaviridae*. Amino acid differences between these partial 3D^pol^ sequences were ≥ 65.7%. Results showed that one bat was co-infected by picornaviruses of more than two genera. Retrovirus, coronavirus, and rotavirus A sequences also were found in the BP sample. The retrovirus and coronavirus genomes were identified in nine and eight bats, respectively. Korean bat retroviruses and coronavirus demonstrated strong genetic relationships with a Chinese bat retrovirus (RfRV) and coronavirus (HKU5-1), respectively. A co-infection was identified in one bat with a retrovirus and a coronavirus. Our results indicate that Korean bats were multiply infected by several mammal viruses.

## Introduction

Bats belong to the order Chiroptera and are mammals with the broadest geographic distribution after rodents^1^. Bats are natural reservoirs for potential zoonotic viruses, such as severe acute respiratory syndrome coronavirus (SARS-CoV), Middle East respiratory syndrome coronavirus (MERS-CoV), coronaviral disease-19, Nipah virus, Hendra virus, and Ebola virus^2,3^. Subsequently, new bat-borne viruses have been continuously discovered around the world: influenza A virus, Phlebovirus, and Banyangvirus^4–6^. Bat viruses are known to have an exceedingly high genetic diversity. In particular, infections of multiple viruses with various genotypes and haplotypes have been reported in an individual bat. Hyperpolymorphic astrovirus haplotypes, which have amino acid differences of approximately 50%, were identified in an individual bat, and the co-infection of coronaviruses with at least two distinct genotypes were also reported in the same bat^7,8^. The wide genetic diversity of bat viruses is known to be due to their unique ecological characteristics, such as frequently high population densities and crowded roosting behaviour^1,9^. Furthermore, in most RNA viruses, low fidelity of the viral polymerase proof-reading function, associated with high replication rate, is known to form a virus haplotype population^10^.

In the last decade, next-generation sequencing (NGS) technology has revolutionised the discovery of novel viruses in humans and animals and expanded our knowledge on viral infection in various mammal species. For example, in serum samples of blood transfusion recipients, a novel human virus that shares genomic features with hepaciviruses and pegiviruses was identified by virome analysis^11^. Additionally, viral diversity from fecal samples of rodents known as natural reservoir of zoonotic viruses, has been characterised, using an unbiased metagenomic approach^12^. In recent years, the importance of bat viruses has also risen rapidly, as bats have been revealed to be natural hosts for viruses that cause several important viral zoonoses, and virome analysis using bat samples have been actively conducted worldwide^13–16^. As a result, a huge amount of genomic sequence data of various viruses from a wide range of bat species has accumulated through virome analysis^17^. As the popularity of virome analysis increases, a database that can analyse large amounts of unknown viral genomes is very important. Although virome analysis is generally based on the NCBI database, it is also critical to use the database depending on the origin of virus. Currently, databases of virus genomes derived from wild animals are represented by DBatVir and DRodVir^17,18^.

In 2015, the first bat-derived viruses in the Republic of Korea (ROK) were discovered from bat fecal samples: coronaviruses, group H rotavirus, bunyavirus, and Banna virus^14^. Following this, alphacoronavirus, betacoronavirus, astrovirus, and paramyxovirus have also recently been reported^7,19,20^. Considering that the viruses detected in bats globally belong to more than 29 families^17^, it is estimated that only a small number of bat viruses have been discovered in the ROK to date. Therefore, in the present study, we confirmed the identity of bat viruses in bat samples from across the ROK by NGS.

## Results

### Next-generation sequencing

Two metagenomic datasets were obtained from a sample of picornavirus-positive bat feces (16BF77) and 74 bat pooling samples (16BP) including 24 bat feces, 25 oral swabs (16BO), and 25 tissues (16BT) collected in South Korea in 2016. Analysis of two metagenomic datasets derived from 16BF77 and 16BP samples generated a total of 24,932,014 and 63,458,357 reads with 71.5% and 79.7% of bases having quality scores ≥ 30, respectively. The clean filtered data was matched with the NCBI and DBatVir databases^17^, and 1877 and 1061 viral reads were identified in 16BF77 and 16BP samples, respectively. In total 28 viral contigs were assembled by GS-assembler from 16BF77, and 201 viral contigs from 16BP, and each contig was annotated by BLASTN. The number of mammalian viruses was 14 contigs in 16BF77 and 29 contigs in 16BP. In the 16BF77 sample, the most common mammal viruses were related to picornaviruses (6 contigs), and in the 16BP sample, several viral sequences were related to coronaviruses (14 contigs), rotaviruses (4 contigs), and retroviruses (3 contigs) (Table 1).

**Table 1:** Composition of the viral sequences in each sample from NGS analyses.

Sample	Family	Virus	No. read	No. contig
**16BF77**				
Phage		Bacterial phage	145	12
Insect virus		Iflavirus	198	2
Mammalian virus	*Picornaviridae*	Picornavirus	1478	6
		Encephlomyocardiosis virus	12	1
	*Retroviridae*	Sarcoma virus	17	2
		Leukemia virus	11	1
	*Herpesviridae*	Herpesvirus	14	3
	*Paramyxoviridae*	Parainfluenza virus	2	1
**16BP**				
Phage		Bacterial phage	858	145
Plant virus		Dwarf virus	51	27
Mammalian virus	*Retroviridae*	Retrovirus	3	3
	*Coronaviridae*	Coronavirus	115	14
	*Picornaviridae*	Encephlomyocardiosis virus	4	1
	*Herpesviridae*	Herpesvirus	5	4
	*Reoviridae*	Rotavirus	12	4
		Banna virus	13	3

### Detection of bat picornaviruses within various genera from 16BF77

**Figure 1:**
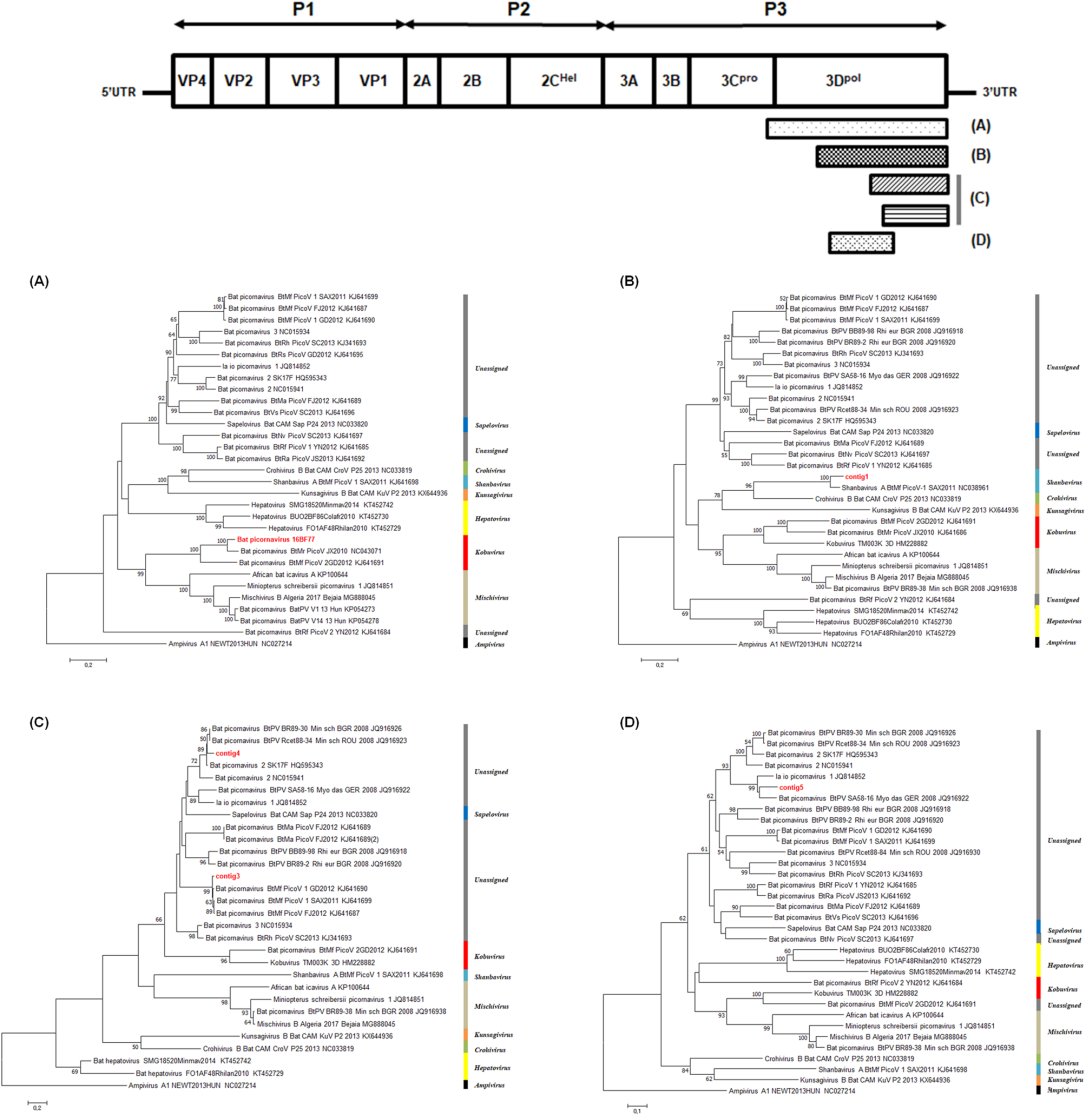
Phylogenetic analyses of bat picornaviruses identified from sample 16BF77. Neighbour joining trees were generated from the final alignments of (**A**) bat PicoV 16BF77 (1188 nt), (**B**) location#16BF77-3 (672 nt), (**C**) location#16BF77-4 and 5 (213 nt), and (**D**) location#16BF77-6 (316 nt), respectively. The numbers at each node indicate bootstrap values as a percentage of 1000 iterations; scale bars indicate the number of nucleotide substitutions per site.

We tested the presence of picornaviruses (PicoVs) in 75 bat samples by RT-PCR. A 217 bp picornaviral genome was detected in a bat fecal sample (16BF77) derived from *Miniopterus schreibersii* collected in Danyang. NGS was performed on the 16BF77 samples to obtain further PicoV sequences. As a result, six picornaviral contigs were obtained and each contig was annotated by BLAST analysis. Each contig had size ranges of 151–894 bp and showed nucleotide similarity of 86.0–98.7% with previously reported bat PicoVs (Supplementary Table S1). Because location#16BF77-1 and 16BF77-2 were matched with the identical reference sequence (BtMr-PicoV/JX2010, NC043071), the sequence gap between the two contigs were filled by specific primers and total of 1474 bp of PicoV sequence (hereafter bat PicoV 16BF77) was obtained (Fig. 1A).

Five PicoV sequences were phylogenetically analysed. Consequently, the sequences were classified into *Kobuvirus*, *Shanbavirus*, and an unassigned group within the family *Picornaviridae* (Fig. 1).

The bat PicoV 16BF77, from location#16BF77-1 and 16BF77-2, showed a similarity of 90.5% with bat PicoV/JX2010 from *Myotis ricketti* (Fig. 1A), and location#16BF77-3 showed a similarity of 88.5% with bat PicoV-1/SAX2011 from *M. fuliginosus* (Fig. 1B). Others also shared high similarities with previously reported bat PicoVs: bat PicoV-1/GD2012 form *M. fuliginosus* (location#16BF77-4; 97.2%), bat PicoV 2/SK17F from *M. magnater* (location#16BF77-5; 91.5%) (Fig. 1C), and bat PicoV SA58-16 from *M. dasycneme* (location#16BF77-6; 84.2%) (Fig. 1D). In addition, the amino acid difference between 16BF77 and location#16BF77-3 was 75.2%.

### Detection and identification of bat retroviruses

**Figure 2:**
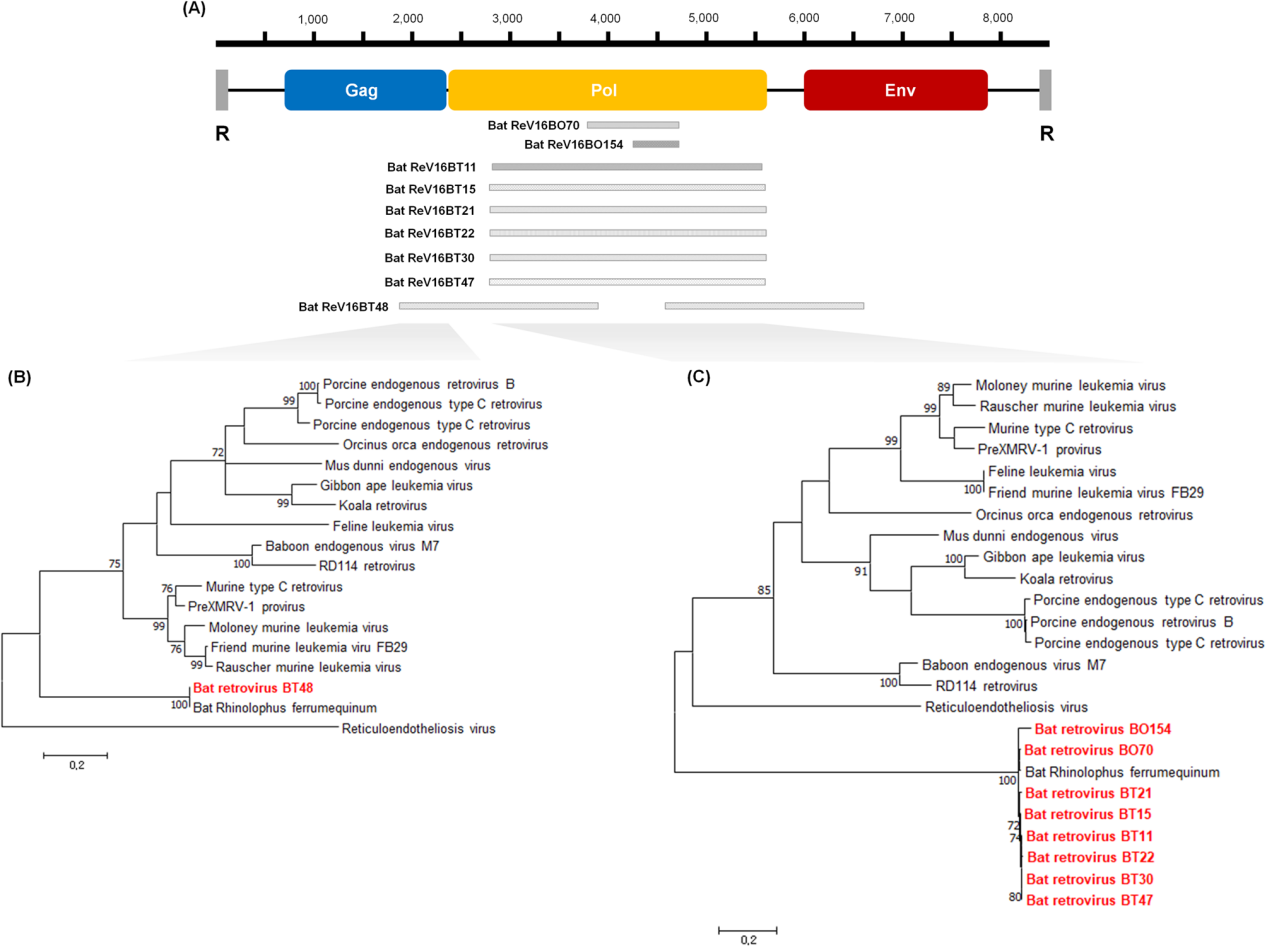
Schematic position of retroviral contigs and phylogenetic analyses of Korean bat retrovirus. (**A**) The obtained Korean bat retroviral sequences were compared to the retrovirus genomic map, and the maximum likelihood trees were constructed using (**B**) a partial *gag* sequence (201 nt) of bat ReV 16BT48, and (**C**) partial *pol* sequences (374 nt) of other Korean bat retroviruses (BO, bat oral swab; BT, bat tissue sample). The numbers at each node indicate bootstrap values as a percentage of 1000 iterations; scale bars indicate the number of nucleotide substitutions per site.

From the 16BP sample, we found three retroviral contigs which corresponded highly with previously reported *Rhinolophus ferrumequinum* retrovirus (RfRV; JQ303225) and the contigs showed similarity ranges of 98.0–98.6% with RfRV (Supplementary Table S3). All bat samples were amplified by a specific PCR based on the contig sequences, and RV sequences were detected from nine *R. ferrumequinum* bat samples (Fig. 2A): seven bat tissue (16BT11, 16BT15. 16BT21, 16BT22, 16BT30, 16BT47, and 16BT48 samples) and two bat oral swab (16BO70 and 16BO154 samples) of *R. ferrumequinum* collected from Gyeongju, Yeongju, Ulju, Sunchang, and Jindo (Supplementary Table S2).

Subsequently, partial retroviral *gag*, *pol*, and *env* genes were further sequenced and phylogenetically analysed with other gammaretroviruses. The partial *pol* gene sequences were obtained from nine of 75 bat samples and the genomic length ranges of each sequence were 330–2697 bp (Supplementary Table S4). The partial *gag* and *env* gene sequences were only identified from a bat tissue sample (16BT48), and sequence lengths were 370 bp and 462 bp, respectively. Partial *pol* sequences, of 374 bp, were phylogenetically analysed, and as a result, the eight bat RVs were clustered with RfRV, with high sequence similarity of 95.2–99.2% (Fig. 2C). In addition, the partial *gag* sequences from 16BT48 also clustered with RfRV with a nucleotide similarity of 99.7% (Fig. 2B).

### Detection and identification of bat coronavirus

Fourteen coronavirus (CoV) contigs were identified from pooled bat samples in this study. The sequences were annotated by BLAST analysis, and were identified to have 90.0–99.1% similarity with Bat-CoV HKU5-1 (EF065509). Each bat sample was tested by a RT-PCR using previously reported pan-CoV primers^21^, which targeted protein RNA-dependent RNA polymerase (RdRp). Bat-CoV sequences were detected from eight bats, which comprised one *Pipistrellus abramus*, one *Eptesicus serotinus*, five *R. ferrumequinum*, and one *Vespertilio sinensis* collected in Andong, Gwangju, Sunchang, and Danyang. Samples consisted of five bat oral swabs, two bat faecal samples, and one bat tissue sample (Supplementary Table S5).

**Figure 3:**
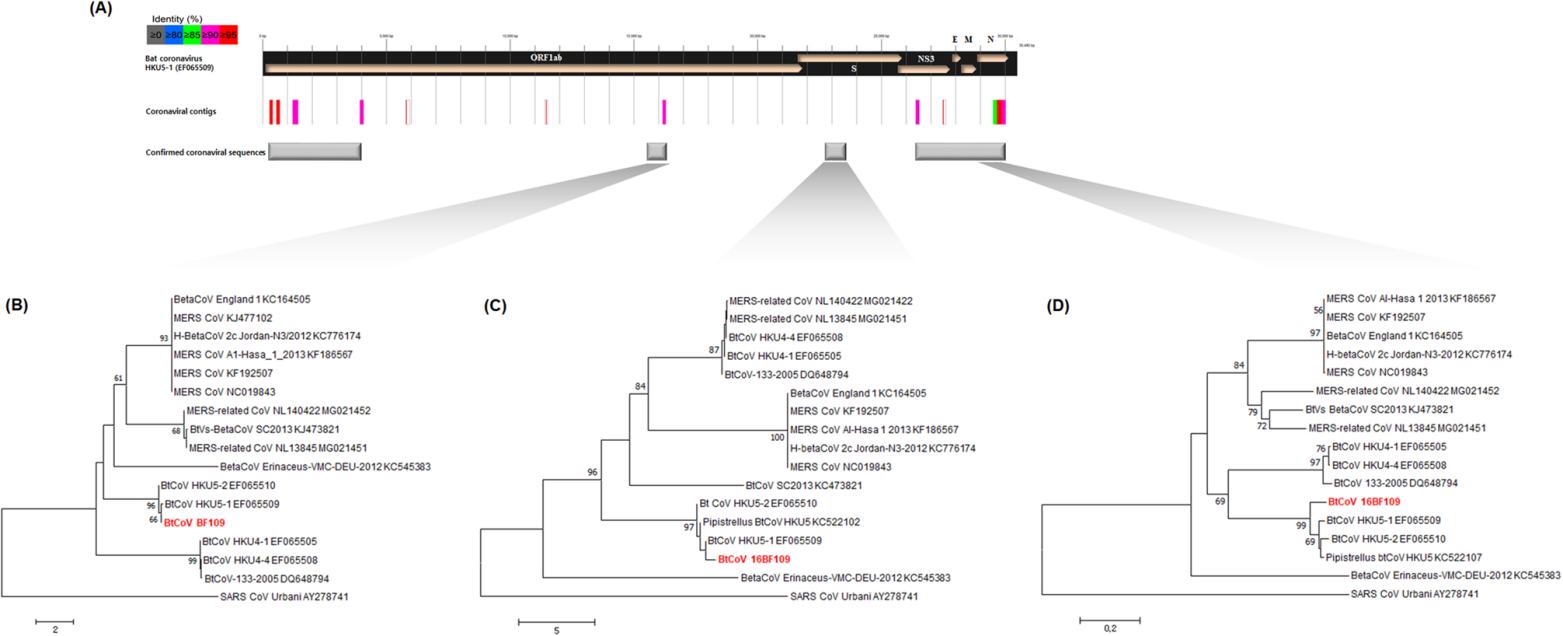
Genomic mapping and phylogenetic analysis of bat coronavirus from sample 16BF109 (BF, bat feces sample). (**A**) The location of the sequences of Bat-CoV 16BF109 were shown by reference to Bat-CoV HKU5-1 (EF065509). Sequence similarities with the reference strain are indicated by different colours and the confirmed coronaviral sequences are marked with grey squares. Neighbour joining trees were generated using (**B**) the partial RdRp (728 nt), (**C**) spike protein (903 nt), and (**D**) NP (835 nt) sequences of Bat-CoV 16BF109. The numbers at each node indicate bootstrap values as a percentage of 1000 iterations; scale bars indicate the number of nucleotide substitutions per site.

Additional coronaviral sequences (5ʹend of ORF 1ab polyprotein, partial RdRp protein, partial spike protein, and NS3d-nucleocapsid phosphoprotein region) were successfully obtained from 16BF109 (*P. abramus*). The phylogenetic analyses of the Bat-CoV 16BF109 using partial RdRp, spike (S), and nucleocapsid phosphoprotein (N) showed a clustering with bat coronavirus Bat-CoV HKU5-1 belonging to the subgenus *Merbecovirus*, within the genus *Betacoronavirus* (Fig. 3). The nucleotide similarities of Bat-CoV 16BF109 were identified to 95.1% (RNA-dependent RNA polymerase, RdRp), 86.4% (Spike protein, S), 95.2% (Envelop protein, E), 93.6% (Membrane protein, M), and 94.0% (Nucleocapsid protein, NP) with Bat-CoV HKU5-1. However, Bat-CoV 16BF109 showed nucleotide similarity of 81.5–84.5% (RdRp), 61.4–63.3% (S), 73.6–76.3% (E), 71.1–73.5% (M), and 74–77.2% (NP) with MERS and MERS-related CoV strains. In addition, the nucleotide similarity ranges were identified to 46–67.3% with SARS CoV Urbani.

### Detection of group A rotaviruses

Four rotavirus contigs with the length ranges of 150–229 bp were detected from bat pooled samples and these contigs were identified as partial VP2, VP3, and VP7 genomes of rotavirus A (RVA) (Supplementary Table S6). Two contigs, location#RVA-1 and 2 showed high nucleotide similarity with VP3 of RVA/Bat/CHN/ZFC23 (MK292705) (97.4% and 97.3%, respectively). These sequences corresponded to positions 2338–2566 nt and 880–1025 nt of the VP3 genomic sequence in strain ZFC23. Location#RVA-3 shared high sequence similarity (96.7%) with the VP2 genomes of RVA/Human-tc/JPN/K8 (JQ713646) and RVA/Rabbit-wt/KOR/rab1404 (MK751433), followed by RAV/Bat/KEN/322/2015 (MH285827). In addition, location#RVA-4 showed the highest similarity (98.6%) with the VP7 genome sequence of RVA/Bat/CHN/LZH and RVA/Bat/CHN/MYAS33 discovered from China, and 98.0% similarity with RVA/Human-wt/CHN/M2-102/2014.

## Discussion

In the present study, NGS was performed to acquire additional PicoV genomes and to identify the presence of bat viruses. We observed various viruses previously unreported in the ROK, and identified picornaviruses belonging to various genera.

PicoVs of the family *Picornaviridae* are non-enveloped positive stranded RNA viruses^22^. The family *Picornaviridae* are a highly diversified virus family with 63 established genera that contain important human and animal pathogens^22,23^. In particular, bat PicoVs were found in various host genera, *Myotis*, *Eidolon*, *Hipposidero*s, *Rhinolophus*, *Coleura*, *Vespertilio*, *Nyctalus*, *Ia*, *Miniopterus*, and *Pipistrellus*. Accordingly, bat PicoVs found worldwide has an extreme genetic diversity and are taxonomically included in various picornavirus genera: *Mischivirus*, *Crohivirus*, *Shanbavirus*, *Kunsagivirus*, *Hepatovirus*, and *Kobuvirus*^13,24–27^.

We found several PicoV sequences encoding the 3D^pol^ gene from the *M. schreibersii* bat (16BF77) through NGS and RT-PCR, and each PicoV sequence showed a sequence similarity of ≥ 85% with previously reported bat PicoV. The high sequence similarities mean that these PicoVs are not novel viruses according to the species demarcation criteria of the family *Picornaviridae* on ICTV^23^. However, these PicoV sequences were included within the genera *Kobuvirus*, *Shanbavirus*, and unclassified groups as a result of our phylogenetic analysis. In addition, the amino acid p-distance was ≥ 65.7% in the analysable areas of these sequences. Although the genomic maps of these PicoVs could not be described by obtaining only partial 3D^pol^ sequences, our results showed that the PicoVs identified in one bat belong to a different genus according to the International Committee on Taxonomy of Viruses (ICTV) genus demarcation criteria of the family *Picornaviridae*: (1) a different genomic structure, (2) significant divergences of ≥ 66% in capsid proteins and ≥ 64% in non-structural proteins^23^. Therefore, these results suggest that bat 16BF77 was multiply infected by various PicoVs belonging to more than two picornavirus genera.

In individual bats, infections of one virus with genetic diversity were often reported. The coexistence of at least two distinct CoV genomes in one bat was reported in Lau et al.^8^ and Chu et al.^28^. In addition, a genetic hyperpolymorphism between astrovirus haplotypes detected from one bat was reported in Lee et al.^7^. In this study, we first identified the multiple infection of viruses of different genera in an individual bat.

RVs (family *Retroviridae*) are positive-sense enveloped RNA viruses with a genome length of 7–12 kb. RVs contain three major proteins: viral capsid (*gag*), protease, reverse transcriptase, and integrase (*pol*), and protective lipid envelope (*env*). The retroviral family consists of seven genera: *Alpharetrovirus*, *Betaretrovirus*, *Gammaretrovirus*, *Deltaretrovirus*, *Epsilonretrovirus*, *Lentivirus*, and *Spumavirus*^29^. Endogenous retroviruses belonging to the genus *Betaretrovirus* were first reported in bats in 2004^30^. Thereafter, diverse groups of endogenous retroviruses, belonging to *Alpharetrovirus*, *Betaretrovirus*, *Gammaretrovirus*, and *Deltaretrovirus*, were identified in megabats and microbats^16,31–33^.

We identified nine bat gammaretroviruses from *R. ferrumequinum* collected in Gyeongju, Yeongju, Ulju, Sunchang, and Jindo. The phylogenetic topologies of these RVs showed a strong genetic relationship with the previously reported RfRV in China. So far, bat gammaretroviruses have been discovered in *Rhinolophus spp*. (*R. pusillus*, *R. pearsoni*, *R. megaphyllus*, and *R. affinis*), *M. ricketti*, *M. natalensis*, and *Pteropus alecto*^32–34^. In the present study, bat gammaretroviruses were only detected in *R. ferrumequinum* among 13 species of Korean bats.

Korean bat gammaretroviruses are regarded as being identical to RfRV, considering their high genetic relationship and a detection in identical host species. Additionally, as a result of verification by PCR in individual samples, the viruses were not only found in pooled tissue samples, including the brain, lung, intestine, and liver, but also oral swabs. Recent studies suggest that RV can bind to and traverse mucosal epithelial cells, and the epithelial cells play an important role in the infection cycle, although they are not targets for viral infection and replication^35^. This indicates that retroviruses can be detected in bat oral swabs, as in the results of the present study. A phylogenetic tree of the *env* protein could not be created because of a divergence among gammaretrovirus sequences. Additionally, two sequences of bat ReV 16BT48 were not connected by specific primers of both sequences and no other RVs could obtain an additional sequence to both ends. Therefore, it is assumed that the bat RVs detected in this study are endogenous viruses within the host genome, not proviruses.

CoVs are enveloped viruses containing the largest (27–32 kb) single stranded positive-sense RNA genome. CoVs belong to the family *Coronaviridae* that contains four genera: *Alphacoronavirus*, *Betacoronavirus*, *Deltacoronavirus*, and *Gammacoronavirus*^36^. In the ROK, Bat-CoVs were first discovered in 2015 from bat faecal samples^14^. Since then, CoVs within genera *Alphacoronavirus* and *Betacoronavirus* have been reported, including SARS-like CoVs and MERS-like CoVs^14,19^. Recently the entire genome sequence for Bat-CoV, which is included in the identical subgenus *Sarbecovirus* as SARS-CoV, has been reported^37^.

The coronaviral contigs obtained through NGS in this study were genetically mapped by matching them with Pipistrellus HKU5-1 CoV, which had the highest frequency in BLAST analysis results. Since the results of the coronavirus prevalence and genetic analysis in some samples used in this study have already been reported^19^, the partial sequence of a spike protein gene was obtained by RT-PCR from bat fecal sample 109 (16BF109) that had genetic differences among the eight positive samples. Our phylogenetic analyses indicated that CoV 16BF109 was included in the subgenus *Merbecovirus*, within the genus *Betacoronavirus*. It confirmed that the Korean Bat-CoV had a high genetic similarity with *Pipistrellus* HKU5-1 CoV, but is genetically distant from the MERS-CoVs. In addition, the Korean Bat-CoVs were found in bats of the family *Rhinolophidae* (*R. ferrumequinum*) and *Vespertiliodae* (*E. serotinus, P. abramus,* and *V. sinensis*)^19^. Since these bat species (*Rhinolophus sp*. and *Pipistrellus *sp.) are also known as natural hosts of SARS-CoV, MERS-CoV, and SARS-CoV-2, which cause severe disease in humans, continuous surveillance of the bat coronaviruses in the ROK is necessary.

RVA is the most representative species in the family *Rotaviridae*, which are known to cause diarrheal disease, mostly from infections in young children and infants^38^. In bats, group A rotavirus in the family *Rotaviridae* has been mainly identified so far, but recently group H rotavirus has also been identified in bats in the ROK and Cameroon^14,39,40^. In the present study, each of the four contigs was identified as partial sequences of VP2, VP3, and VP7 segments of the RVA genome. The partial sequences of VP3 and VP7 segments appeared to have high sequence similarity with previously reported bat RVA (bat RVAs ZFC23 and LZHP2), particularly, while the VP7 gene was most similar to bat RVA LZHP2, which belongs to the G3 genotype. Therefore, the Korean bat RVA is presumed to belong to the G3 genotype. The partial sequences of VP2 and VP7 segments were most similar to human and rabbit-originated RVA and bat-originated RVA, respectively. In particular, the bat-originated LZHP2 strain provides strong evidence of cross-species transmission between bats and human^41^. Although we conducted a partial genetic analysis, it is assumed that cross-species transmission or dynamic reassortment has occurred in Korean bat RVA, considering the high genetic similarities with various host-derived RVAs. However, it was not possible to confirm whether the sequences of each rotavirus segment derived from one virus because no rotavirus was detected for individual bats by PCR through the gap filling of partial VP3 genes. Further analysis of recombination events and cross-species transmission by obtaining additional sequences in the Korean bat RVA are needed.

In our surveillance study, the co-infection of bat RVs and CoVs were identified in the oral swab sample of an *R. ferrumequinum* individual collected in Sunchang. In addition, bat 16BF77 was confirmed to have been infected with more than two picornavirus genera through NGS. Indeed, there have been reports of co-infections of two different viruses in one bat before: co-infection of CoV and AstV^42^, and of CoV and paramyxovirus^43^. The co-infection of more than two viruses in a single host often causes genetic recombination, such as heterologous inter-family recombination as reported by Huang et al*.*^44^. This can be explained by the widely accepted recombination model copy-choice recombination, which is mediated by template switching in the course of RNA synthesis during co-infection with two viruses^45^. It is speculated that such frequent recombination eventually increases the genetic diversity of bat viruses and the potential possibility for transmission to other hosts. Furthermore, we report the first identification of the co-infection of various *Picornavirus* genera in one bat, and we cautiously speculate that these results could be one cause of the extreme genetic diversity of the picornaviridae viruses.

Only a small number of bat viruses have been detected in the ROK, compared to more than ten thousand bat viral genomes recorded recorded world^17^. Although several viruses have been identified in Korean bats, these viruses showed the closest genetic relationship with bat viruses reported in China. It is speculated that there might have been interactions between Chinese and Korean bat populations, because the ROK is geographical neighbors with China. In practice, bats are the only mammals capable of flight and bats of many species fly long distances; *Myotis spp*. are known to fly up to 400 miles^1^.

In conclusion, we provide the first report identifying bat PicoVs and RVs in the ROK, and confirm the co-infections of various PicoVs belonging to more than two genera in one bat. We also identified the co-infection of RV and CoV in an individual bat. As the existence of more diverse viruses is supposed in Korean bats, continuous surveillance will be necessary to detect potential zoonotic viruses.

## Materials and methods

### Bat samples

**Figure 4:**
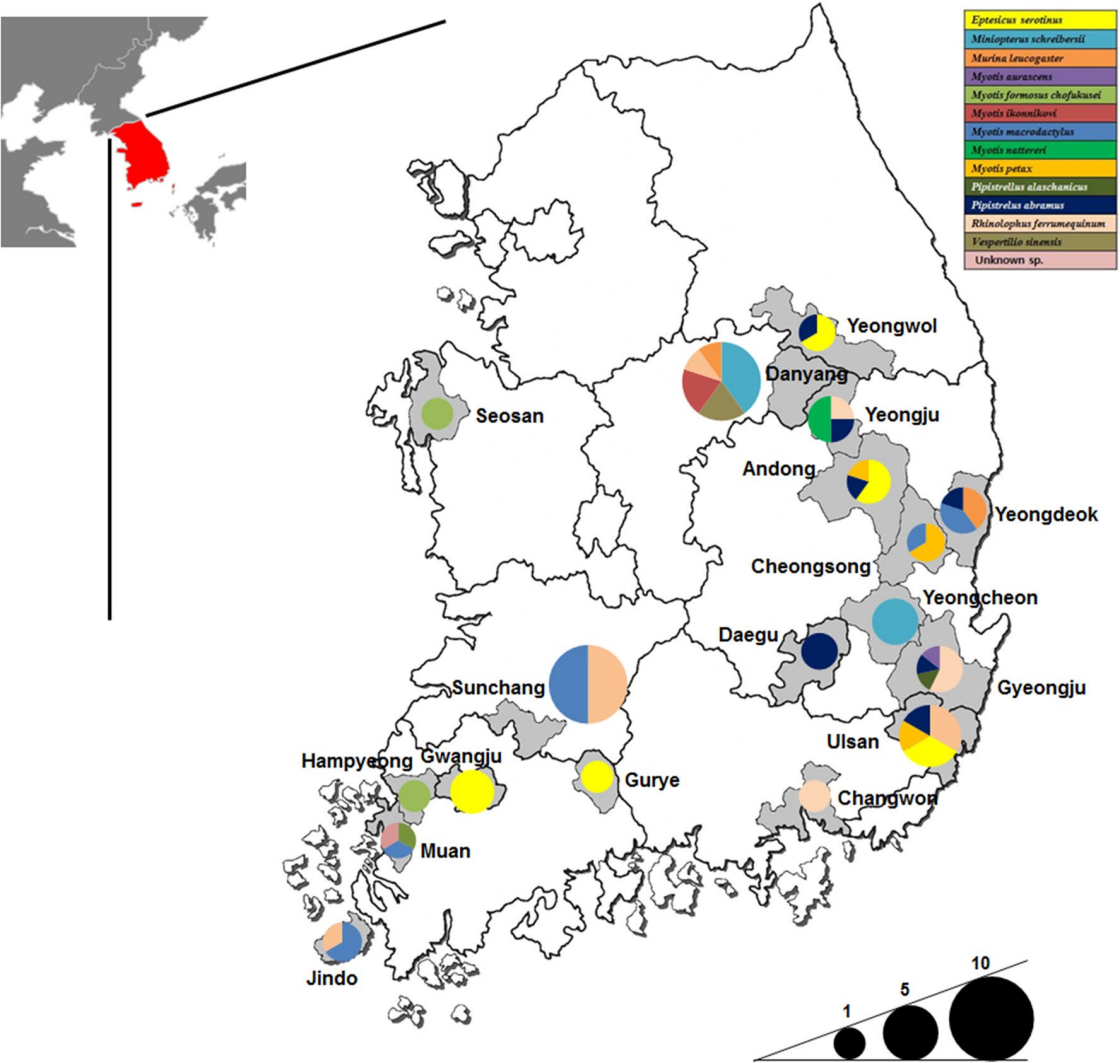
Geographic location of sampling sites. Thirteen bat species are indicated in different colours, and collected bat species are indicated by colour fractions in each circle. The size of the circles indicates the number of collected bat samples in each province in the ROK (Adobe illustrator CS6, www.adobe.com).

A total of 75 bat samples (25 faecal, tissue, and oral swab samples, respectively) were collected from 13 different bat species from 18 provinces in the ROK, 2016 (Fig. 4). Oral swabs were transported in viral transport medium (Copan Diagnostics, CA, USA). Bat carcasses were autopsied for collection of internal organ tissues, including brain, lung, intestine, and liver tissues, in the biosafety laboratory-2 (BSL-2) cabinet. All collected organ tissues were pooled and ground in phosphate-buffered saline containing 1% antibiotic–antimycotic solution (anti-PBS) (Corning, USA). Bat faecal samples were vigorously vortexed in 1 mL anti-PBS. All samples were stored at − 70 ℃ until used.

### Detection of picornaviruses

Total RNA was extracted from 75 bat samples using the QIAmp viral Mini Kit (Qiagen, Hilden, Germany) and random cDNA was synthesised using the SuperScript III First-Strand Synthesis Kit (Invitrogen, Carlsbad, CA, USA) according to the manufacturer’s instructions. Picornaviral genome detection was performed by hemi-nested PCR (using primers Bat-PV1: GGTGGYAATCCTTCTGG and Bat-PVR: TTTGTCAGGGGGAGTCA for the first PCR, and Bat-PV2: GTACCACTGTTTTCAAT and Bat-PVR for the second PCR) targeted a 3D^pol^ gene in PicoVs, which has a length of 217 bp. Amplification was performed using the Platinum Green Hot Start PCR Master mix (Invitrogen, Carlsbad, CA, USA) according to the manufacturer’s protocols. Amplification conditions are as follows: initial incubation at 94 ℃ for 1 min, followed by 40 cycles of denaturation 94 ℃ for 30 s, annealing at 50 ℃ for 30 s, and extension at 72 ℃ 30 s, with a final extension at 72 ℃ for 5 min in a Mastercycler (Eppendorf, Hamburg, Germany). PCR product was confirmed by the ABI 3130 sequencer (Applied Biosystems, Foster City, CA, USA) to be a picornaviral genome.

### Sample preparation

Two bat samples were prepared for NGS, which were a pool of 74 bat samples (BP) for virome analysis and a PicoV-positive sample (16BF77) for further sequencing. To remove host cells and other debris, fluids of each sample were centrifuged at 18,000*g* rpm for 10 min at 4 ℃. For the virome analysis, each sample was collected in 300 µL and pooled supernatant was filtered through a 0.22 µm membrane filter (Millipore, Darmstadt, Germany) for elimination of eukaryotic cells and bacteria. For the PicoV-positive sample, 2 mL of supernatant was filtered as mentioned above. Each 200 µL of filtered fluid was used for total viral RNA extraction using a QIAamp viral RNA Mini Kit (Qiagen, Germany).

### Library, sequencing and assembly

cDNA was synthesised using a Superscrip II cDNA Synthesis Kit (Invitrogen, Carlsbad, USA) and a SMARTer PCR cDNA Synthesis Kit (Clontech, CA, USA) according to the manufacturer’s instructions. The cDNA library was produced by an Illumina TruSeq RNA Library Prep Kit (Illumina, CA, USA). For cDNA concentrations and library length measurements, LightCycle qPCR (Roche, Penzberg, Upper Bavaria, Germany) and Agilent High Sensitivity D1000 ScreenTape system (Santa Clara, CA, USA) were used, respectively. The quantified libraries were amplified to cluster on the flow cell and sequenced using an Illumina Hiseq 4000 instrument as recommended by the manufacturer. Sequencing reads were quality-trimmed using Trim Galore! (Babraham Bioinformatics, UK) and classified by DconSeq using a database created from the National Center for Biotechnology Information (NCBI) and DBatVir. Following contig assembly by GS-assembler and CAP3 program, viral contig annotations were selected by BLASTn.

### Detection of viral genome and gap filling

To confirm the presence of RV and RVA, specific primer sets were designed based on the viral contig sequences obtained by NGS. For CoV, two primer sets were synthesised: previously reported primers for the detection of the RdRp gene^21^ and designed primers from the alignment of spike protein sequences. In addition, the shredded viral sequences from NGS were concatenated by a primer designed based on the metagenomic sequencing results obtained in this study (Supplementary Table S7). Extraction and amplification of the total RNA from each individual sample was performed as previously described by Lee et al*.*^7^. Amplification conditions were as follows: initial incubation at 94 ℃ for 1 min, followed by 35 cycles of denaturation 94 ℃ for 30 s, annealing at 50–58 ℃ for 30 s, and extension at 72 ℃ for 30 s, with a final extension at 72 ℃ for 5 min in a Mastercycler (Eppendorf, Hamburg, Germany). PCR product was sequenced using an ABI 3130 analyzer (Applied Biosystems, Foster City, CA, USA).

### Phylogenetic analysis

Phylogenetic analysis was carried out based on the obtained viral sequences from individual samples. The viral sequences for analyses were retrieved from the GenBank database. Multiple alignments of viral sequences were generated using the Clustal W method in BioEdit version 7.0.9.0. Phylogenetic trees were generated by maximum-likelihood (ML) and neighbour-joining (NJ) methods using MEGA7.0 (Molecular Evolutionary Genetics Analysis 7.0) software^46^. NJ tree topologies were evaluated by 1000 bootstrap iterations using MEGA7.0.

### Ethics declarations

All experiments were carried out in accordance with relevant guidelines and regulation and approved by the ethic committee of Jeonbuk national university.

## Supplementary information

Supplementary Information.

## Data Availability

The Illumina Hiseq 4000 data have been deposited in the GenBank Sequence Reads Archive (SRA) under accession number PRJNA637211 and all consensus virus genome sequences have been deposited in GenBank under accession numbers MT648449-MT648461.
